# Quality of life and mental health in hemodialysis patients: a challenge for multiprofessional practices

**DOI:** 10.1590/2175-8239-JBN-2018-0227

**Published:** 2019-01-21

**Authors:** Debora Berger Schmidt

**Affiliations:** 1 Pró-Renal Brasil, Curitiba, PR, Brasil.

Chronic Kidney Disease (CKD) could represent all the current problems we have in healthcare: if, on the one hand, treatment advances enable better health maintenance and life extension, on the other hand they do not necessarily guarantee quality improvements. Although the achievements and progressive advances in the treatment of people with renal disease are evident, several studies reinforce the prevalence of neuropsychopathologies associated with CKD treatment - such as depression, anxiety disorders, cognitive functioning impairment, fatigue, among others - all easily recognized among the complaints and adaptive difficulties reported by patients and their relatives during disease management. Given this, it is important to consider that the illness causes significant changes that require the adaptation of those who experience it, and can influence the way in which the person perceives and qualifies his life.

The World Health Organization Quality of Life Group defines the term Quality of Life (QOL) as: "The individual's perception of his situation in life in the context of the culture and value system in which he lives and in relation to his goals, expectations, standards and concerns."^2^ Since a person's perception of one's routine is individual, QOL is subjective and multidimensional, considering physical, emotional, social, cultural and family standards, among others. In a Brazilian study^3^ on QOV involving286 people in hemodialysis, the mean score was 60.53 (± 14.10), scores lower than those found in US (63.7) and European (62.7) studies. The results lead to the reflection about the factors most perceived as impacted by the disease and the treatment, enabling the planning of therapies that include subjective aspects to improve quality of life.

Depression refers to the most frequently described psychiatric disorder in patients with chronic kidney disease, with a prevalence rate of 20% to 30% in hemodialysis patients. Advanced CKD stages are associated with the high prevalence of previously undiagnosed psychological and psychiatric disorders and quality of life. Depression in hemodialysis patients is associated with high morbidity and mortality, reduced treatment compliance and worsening nutritional status.^1^ Although very common in patients with CKD, depression is underdiagnosed, and its management is challenging, since somatic complaints associated with CKD mimic depression symptoms (such as fatigue, anorexia, weight changes, sleep disorders, nausea and pain).^1^

In addition, limited compliance towards psychiatric treatments by these patients should be considered, as they often complain of numerous parallel treatments and multidrug use. The patient's difficulty in joining out-of-site appointments and dialysis hours also corroborates that mental health-focused treatments take second place, requiring health professionals to make an interdisciplinary effort that encompasses the very complexity of the patient and his treatment. It is in this context that multiprofessional therapies, especially non-pharmacological ones, are consolidated as important practices for approaching mental health in patients with renal disease, making music therapy one of the possible approaches for patients with depression, who can benefit from interventions during the treatment.

The article by Hagemanne et al.,^4^ entitled "The effect of music therapy on quality of life and on the symptoms of depression in patients under hemodialysis", evidences music therapy as an effective option in the treatment and prevention of depression symptoms and in the improvement of QOL in patients in hemodialysis. In the study, the researchers assessed 23 patients before and after eight sessions of 75 minutes of music therapy during the hemodialysis session. The assessment included a quality of life questionnaire (KDQOL-SF) and an instrument for the presence of depressive symptoms (Beck Depression Inventory - BDI-II), and the comparative results of the evaluations indicated that the participants had statistically significant improvement in generic dimensions of (*p* = 0.011) of QOL, as well as in specific dimensions associated with CKD: functional capacity (*p* = 0.004), pain (*p* = 0.036), general health status (*p* = 0.01), vitality (*p* = 0.004), mental health (*p* = 0.012), list of symptoms and problems (*p* = 0.01) and overall health (*p* = 0.01). The patients presented a significant reduction in the depression symptoms (*p* < 0.001).

The study corroborates the study by Pivatto et al.,^5^ who evaluated the impact of music therapy on hemodialysis patients, noting that there was a 75% decrease in problems during the sessions in which the music therapy activities occurred when compared to the sessions without such intervention. Patients also reported that perception of the passage of time was understood to be accelerated in 79% of the patients and there was a prevalence of positive feelings during the HD sessions composed by music therapy interventions.

It should be emphasized that music therapy has been inserted in several areas in healthcare, and in hemodialysis is going through a path of consolidation as a practice of promoting quality of life, encouraging an active involvement of patients.^4^ It is a practice of care that contributes to the feeling of welcoming, belonging and expanding the possibilities of coping.

In general, quality of life surveys help us understand which aspects are involved and impacted in each health condition, and the study in question enables the idea that interdisciplinarity is a additional way to address the complexity of chronic kidney disease, bringing up the importance of practices that address depression and promote the quality of life in an effective way, integrated to the routine of patient care.
